# Polarized white light from hybrid organic/III-nitrides grating structures

**DOI:** 10.1038/srep39677

**Published:** 2017-01-03

**Authors:** M. Athanasiou, R. M. Smith, S. Ghataora, T. Wang

**Affiliations:** 1 Department of Electronic and Electrical Engineering, University of Sheffield, United Kingdom

## Abstract

Highly polarised white light emission from a hybrid organic/inorganic device has been achieved. The hybrid devices are fabricated by means of combining blue InGaN-based multiple quantum wells (MQWs) with a one-dimensional (1D) grating structure and down-conversion F8BT yellow light emitting polymer. The 1D grating structure converts the blue emission from unpolarised to highly polarised; Highly polarised yellow emission has been achieved from the F8BT polymer filled and aligned along the periodic nano-channels of the grating structure as a result of enhanced nano-confinement. Optical polarization measurements show that our device demonstrates a polarization degree of up to 43% for the smallest nano-channel width. Furthermore, the hybrid device with such a grating structure allows us to achieve an optimum relative orientation between the dipoles in the donor (i.e., InGaN/GaN MQWs) and the diploes in the acceptor (i.e., the F8BT), maximizing the efficiency of non-radiative energy transfer (NRET) between the donor and the acceptor. Time–resolved micro photoluminescence measurements show a 2.5 times enhancement in the NRET efficiency, giving a maximal NRET efficiency of 90%. It is worth highlighting that the approach developed paves the way for the fabrication of highly polarized white light emitters.

The last twenty years have seen unprecedented progress in developing white light emitting diodes (LEDs) based on III-nitride blue LEDs^1^, leading to the solid-state lighting revolution and massive energy savings. So far, a combination of a blue emitting InGaN LED with a down-conversion yellow phosphor still remains the major approach to achieving white light. However, this technique has a number of drawbacks, such as self-absorption induced poor conversion efficiency from blue to yellow and a low light quality leading to serious issues of human sleep patterns and thus mental health^2^. Furthermore, such an approach generates significant limitations to areas such as display backlighting, where polarized white light is required and visible light communication applications where bandwidth is limited by the intrinsically slow response time of yellow phosphors.

A number of approaches have been proposed in order to address these challenging issues, for instance, monolithically integrated white light LEDs^3^,^4^, hybrid III-nitride/colloidal quantum dots^5^,^6^ and hybrid III-nitride/organic conjugated polymers^7^,^8^. An ideal solution would be a monolithically grown white light LEDs with all emission components (at least blue and yellow) exhibiting both polarized emission properties and high efficiency. Currently, such an approach would be impossible as it requires high efficiency yellow LEDs with a high degree of optical polarization and the yellow emission which further needs to be match the alignment of the polarized blue emission. Due to the well-known quantum confined stark effect (QCSE)^9^,^10^, the growth of longer emission wavelength LEDs beyond the blue spectral region tends to be very difficult, leading to significantly reduced quantum efficiency and thus making the approach impossible at the moment. Furthermore, the emission from any standard c-plane LEDs is intrinsically unpolarised. A combination of CdSe/ZnS colloidal quantum dots with III-nitride blue LEDs has been used to achieve high performance white light sources demonstrating excellent CIE coordinates with high color rendering index (CRI)^10^,^11^, although new regulations recently launched poses strict limitations to the usage of Cd-based quantum dots as a result of their toxicity which can be potentially hazardous to human health. Furthermore, this method still faces significant challenges in achieving polarized white light emission.

Compared with existing phosphors or colloidal quantum dots, light emitting polymers as down-conversion materials have a number of major advantages. They can have high photoluminescence efficiency in the longer wavelength emission spectral regions, such as yellow. It is well-known that that light emitting polymers generally exhibit a large Stokes-shift, which can effectively eliminate the self-absorption issue. In addition, the good dissolvability of light emitting polymers in a solvent such as toluene can simplify the fabrication process of hybrid organic /inorganic nanostructures by just employing standard spin-coating techniques. The most important feature for such a hybrid white LED using a light emitting polymer as a down conversion material is due to significant potentials to enhance the efficiency of color conversion, which can be achieved through a non-radiative resonant energy transfer (NRET) process, namely, non-radiative Förster energy transfer^5^,^12^,^13^,^14^. In this case, the color quality of such a hybrid white LED can be significantly improved compared with current white LEDs fabricated using yellow phosphors.

Furthermore, the utilization of light emitting conjugated polymers is particularly important for the fabrication of polarized emitters, making use of another major advantage that liquid crystal (LC) phases can exist due to the conjugated backbone and long alkyl side chains^12^. Liquid-crystal polymers naturally exhibit a preferential alignment in a microscopic domain, demonstrating unique properties such high carrier mobility^15^,^16^ and polarized light emission^12^,^17^. By controlling the alignment properties from microscopic to macroscopic domains, polarized yellow light could be effectively produced^18^,^19^.

A further requirement is that we need to convert unpolarised blue emission from a standard c-plane InGaN/GaN multiple quantum well (MQW) structure into polarized emission. Fabrication of the MQW into a one dimensional (1D) grating structure is a promising approach. In this case, such a grating structure also serves as a polarizer and thus could emit polarized light.

By means of combining such III-nitride structures with the conjugated polymers in a compatible manner, a hybrid organic/inorganic structure with highly polarized white light emission with high CRI can potentially be achieved.

A number of approaches have been explored to align conjugated polymers such as F8BT chains, for example, nanoimprinting techniques^15^,^18^,^20^, electro-spinning fabrication techniques^21^,^22^ and thermomechanical alignment of polymers^23^. In this work, we report a different but efficient nanofabrication technique for the fabrication of a 1D grating structure on a standard InGaN/GaN MQW structure which can convert unpolarized blue light to polarized light as a result of the 1D grating structure. Such a 1D grating structure consists of a large number of periodic nano-channels, into which the yellow emitting F8BT polymer can be filled and these nano-channels can then be further used to accurately align the F8BT chains in macroscopic domains for the generation of polarized yellow emission.

Such a hybrid structure is also expected to significantly enhance the NRET process between the donor (InGaN/GaN MQWs) and the acceptor (F8BT) due to dipole-dipole coulombic interactions. As a result of the NRET process, a reduction in non-radiative recombination in the donor is also expected due to the extra channel for the excitons in the donor to migrate into the acceptor. The NRET process sensitively depends on the dipole-dipole separation, typically <10 nm^14^,^24^. Furthermore, it is also sensitive to not only the relative orientation of the two kinds of dipoles (namely, the diploes in a donor and the diploes in an acceptor) but also the spectral overlap between the emission from the diploes in the donor and the absorption from the diploes in the acceptor. Optimizing these parameters could potentially lead to a significant enhancement in NRET which in turn could dramatically increase the efficiency of white light emission. A significant enhancement in NRET process has been confirmed by our group by means of fabricating a hybrid III-nitride nanorod/F8BT device as a result of minimizing the separation of the two kinds of dipoles^14^,^25^. However, due to the random nature of the formation of III-nitride nanorods and the random distribution of the F8BT polymers used, polarized white cannot be achieved.

In this work, as mentioned above, hybrid F8BT polymer/1D III-nitride grating structures containing periodic nano-channels have been fabricated on a standard c-plane InGaN/GaN MQW structure, where the F8BT can be effectively filled into the nano-channels. Such a hybrid configuration can allow us to effectively manipulate the relative orientation of the dipoles in the donor and the diploes in the acceptor in order to produce polarized emission in a compatible manner. This approach can also lead to further enhancement in NRET between the dipoles in the donor and dipoles in the acceptor. As a result, a NRET efficiency of up to 90% has been achieved when the dipoles in the donor and the dipole in the acceptor are aligned in a parallel orientation. The resultant generation of highly efficient and polarized white light is particularly useful for backlighting applications.

A standard 5 period InGaN/GaN MQW *epi-wafer* is used in this work, where each InGaN quantum well with a thickness of 2.5 nm and ~20% indium content is sandwiched by 10 nm GaN barriers. The sample was grown on a *c-plane* sapphire substrate using a metal organic chemical vapour deposition (MOCVD) system. A commercial confocal microscope system equipped with a 375 nm diode laser has been modified in order to write a 1D grating mask on a nanometre scale on the InGaN/GaN MQW structure (see supplementary information for the details of fabrication). A set of nine grating structures with different nano-channel widths, varying from 43 nm to 294 nm, have been fabricated in order to study the dipole alignment as a function of nano-channel width. The centre to centre separation was kept constant at 510 nm. Figure 1a to i show the top-view scanning electron microscopy (SEM) images of these grating structures with different nano-channel widths. For the fabrication of the hybrid structures, the F8BT polymer was dissolved in toluene with a concentration of 5 mg per mL, followed by spin-coating it on the top of the grating structures in order to fill into the nano-channels. Such a deposition of the F8BT by the spin coating method yields a random alignment of the F8BT chains even though the F8BT is filled into the nano-channels. As a result, the random alignment of the F8BT chains does not lead to an effective NRET process between the dipoles in the donor (i.e., the InGaN/GaN MQWs) and the dipoles in the acceptor (i.e., the F8BT). In order to produce the best alignment of the F8BT chains along the nano-channel orientation, the hybrid samples were heated up to 160 °C, above the glass transition temperature of the F8BT polymer (T_g_ ~ 90 °C), allowing the F8BT to transform into a LC phase. In order to further enhance the alignment of the F8BT chains, a high pressure was simultaneously applied to the hybrid sample so that the F8BT chains can be well-aligned along the nano-channel orientation in a controlled and best manner (see supplementary information for detail fabrication procedure).

The optical properties of all the 1D grating structures have been investigated as a function of nano-channel width by means of micro-photoluminescence (μ-PL) measurements, which have also been compared with the un-patterned sample. Our μ-PL system is equipped with a 375 nm pulsed laser diode as an excitation source. For the polarisation dependent PL measurements, a polariser was placed before the collection fibre in order to investigate the polarisation properties of these samples. As an example, Fig. 2a shows the PL spectra of one 1D grating structure with a nano-channel width of 294 nm as function of polarisation angle from zero to 360°, where for comparison the inset gives the polarisation angle dependent PL spectra of the un-patterned sample. As expected, all the PL spectra of the un-patterned samples remain unchanged due to the un-polarised nature of the emission from the c-plane InGaN/GaN MQW structure. In remarkable contrast, a clear intensity variation and a change in emission peak wavelength from the grating structure have been observed as a function of polarisation angle as show. Figure 2b and c present the integrated PL intensity and the emission wavelength as a function of polarisation angle in the polar coordinate system, respectively, demonstrating clear polarised emission, while the emission from the un-patterned sample does not exhibit any polarisation as a result of its intrinsic isotropic strain. A strain relaxation can be introduced through fabrication into nanostructures, as it has been previously studied^9^,^10^,^26^. In the case of a grating structure, the 1D configuration leads to anisotropic strain relaxation, as the strain relaxation takes place only in a direction which is perpendicular to the grating orientation while the strain along the grating orientation remains under its intrinsic strain conditions. As a result of the anisotropic strain relaxation, two optical transitions exist due to splitting of the valence band into two distinct subbands^27^,^28^ leading to the optical polarisation as observed in Fig. 2a. The anisotropic strain relaxation also causes dipoles in the donor (i.e., the InGaN MQWs) to be preferentially orientated along the grating direction. For the unpatterned sample, the emission peak remains unchanged against the polariser angle as expected, because the emission from any c-plane InGaN MQWs is naturally unpolarised and the dipoles are randomly aligned within the in-plane quantum wells.

Polarisation dependent PL measurements have also been performed on our hybrid samples using the μ-PL system. As an example, Fig. 3a shows typical spectra of our hybrid structure with a nano-channel width of 43 nm, measured at the two extreme polarisation angles, namely, one with a polariser placed in parallel with the grating orientation and another measured with the polariser along the perpendicular direction. The inset of Fig. 3a shows the integrated intensity of the emission from the MQWs, the F8BT and the overall emission including both the blue emission and the yellow emission, respectively, as a function of polarisation angle plotted in the polar coordinate system. Figure 3b presents the polarisation degrees of all hybrid samples as a function of nano-channel width, where the polarization degree can be calculated by Equation 1 below


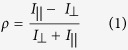


where I_⊥_ and *I*_*||*_ are the integrated PL intensities measured along the direction which is perpendicular to the grating orientation and another parallel to the grating orientation^29^, respectively.

Figures 3b shows the polarisation degree of the emission from the InGaN/GaN MQWs and the yellow emission from the F8BT as a function of nano-channel width, respectively. A monotonic decrease in polarisation degree has been observed, from 68% to 40% for the MQWs with reducing nano-channel width. The polarization of the emission from the c-plane InGaN/GaN MQWs is generated due to anisotropic strain relaxation^27^ as a result of being fabricated into a grating structure featured with nano-channels. With increasing nano-channel width, the anisotropic strain relaxation is enhanced, leading to an increase in polarization degree^27^ Furthermore, it is also well-known that the effective refractive index of a grating structure decreases with reducing the fill factor of the grating structure (namely, increasing nano-channel width in our case), leading to an increase reduction in reflection and thus an increase in polarization degree^30^. On the other hand, the polarisation degree of the F8BT gradually increases with decreasing nano-channel width. The highest polarisation degree of the emission from the F8BT was 48%, giving an overall polarisation degree of 41% for the whole emission (including both blue and yellow). This suggests that a reduction in nano-channel width enhances the confinement of the F8BT chains on a nanometre scale^18^, leading to better alignment of the polymer along the grating orientation and thus enhancing the alignment of the dipoles in the F8BT chains^31^. In order to further test this argument, an identical set of hybrid grating samples were fabricated for comparison. For these reference samples, the F8BT polymer which is filled into the nano-channels is not subject to any macroscopic alignment process mentioned above. Polarisation dependent PL measurements have been performed on these reference samples, all exhibiting zero polarisation degree of the F8BT regardless of nano-channel width as a result of hoping effect, namely, the excitons in the F8BT can hop into the neighbouring chains with different orientations^15^,^16^,^17^,^18^. Fig. 3b also includes a typical example of the reference, labelled in red.

Exciton recombination dynamics have been investigated on the hybrid structures in order to study the influence of the relative orientation between the dipole in the donor and the dipoles in the acceptor on the NRET process. Time resolved micro-photoluminescence (μ-TRPL) measurements have been performed at room temperature using a time-correlated single photon counting (TCSPC) system combined with a Horiba IHR550 spectrometer. A 375 nm pulsed laser diode with a 50 picoseconds (ps) pulse width was used as an excitation source. For comparison, an identical set of grating structures but filled with PMMA organic polymer have been fabricated, where the absorption wavelength of the PMMA does not match the emission wavelength of the InGaN MQWs and thus there is no NRET process between them.

Figure 4a schematically illustrates the NRET mechanism between the donor (i.e., InGaN MQWs) and the acceptor (i.e., F8BT) dipoles, along with a relaxation process generating photons whose energy is equal to the bandgap energy of the F8BT polymer.

As an example, Fig. 4b shows the PL decay trace of the reference sample (i.e., coated with PMMA, and thus there is no NRET) and our hybrid sample (i.e., filled with the F8BT) with a nano-channel width of 43 nm. The PL decay curves have been measured at the peak wavelength of the InGaN MQWs, which is 460 nm. Figure 4b demonstrates a significantly reduced PL decay lifetime for the hybrid sample compared with the reference sample.

For the reference sample, the PL recombination decay rate is given by Equation 2


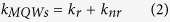


where *k*_*MQWs*_, *k*_*r*_ and *k*_*nr*_ are the total, radiative and non-radiative decay rates, respective.

For the hybrid structures, the exciton recombination dynamics have to be modified as a result of the NRET process taking place between the InGaN MQWs and the F8BT polymer as discussed in the introduction section. Therefore, the PL decay rate is modified to Equation 3


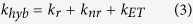


where *k*_*ET*_ is the non-radiative energy transfer rate (*NRET*). A bi-exponential model can be used to fit the PL decay traces based on Equation 4 below^32^


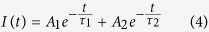


where A_1_ and τ_1_ (A_2_ and τ_2_) represent the fast (slow) decay components, respectively.

Figure 4c shows the extracted PL lifetimes plotted as a function of nano-channel width. For the reference sample, the PL lifetime is fairly constant regardless of the nanochannel width. In remarkable contrast, the hybrid samples show a monotonic reduction in PL lifetime with reducing nano-channel width when the nano-channel width is below 100 nm in size, as shown in Fig. 4c.

The NRET rate between two dipoles can be described by Equation 5^33^,^34^


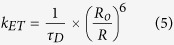


where *τ*_*D*_ is the decay lifetime of the donor dipole; *R* is the distance between the dipoles in the donor and dipoles in an acceptor; and *R*_o_ is the Förster distance between the dipoles in the donor and dipoles in the acceptor at which the transfer efficiency is 50%. Moreover, the Förster distance is given by Equation 6^33^,^34^


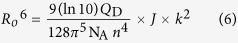


where *Q*_D_ is the quantum yield of the donor; *N*_*A*_ is Avogadro’s number; *n* is the refractive index of the medium where the non-radiative Förster energy transfer takes place; *J* is the overlap between the emission spectrum of the dipoles in the donors and the absorption spectrum of the dipoles in the acceptor; and *k* is the dipole orientation factor. The above Equations 5 and 6 clearly show that the NRET rate strongly depends on the dipole orientation factor of the two kinds of dipoles (i.e., the dipoles in the donor and the dipoles in the acceptor, respectively) in addition to the spectral overlap and the distance of the two kinds of dipoles.

In our case, both the distance between the two kinds of dipoles and their spectral overlap remains identical for all the samples. However, the dipole orientation factor (*k*^2^) can be modified through tuning the nano-channel width as discussed above and can take a value in the range from 0 to 4^33^,^34^. For instance, when the two kinds of dipoles are perpendicular to each other, the dipole orientation factor is zero (*k*^2^ = 0), thus prohibiting any energy transfer between them. If the two kinds of dipoles are randomly oriented, an average value for *k*^2^ = 2/3 can used, as the relative orientation between the dipoles in the donor and the diploes in the acceptor are random. The maximum NRET rate can be achieved when the dipoles in the donor and the dipoles in the acceptor are in parallel, leading to *k*^2^ = 4^33^,^34^. The NRET process can be enhanced by optimising the alignment of these two kinds of dipoles through tuning the nano-channel width as we observed when the nano-channel width is below 100 nm. In this case, the dipoles in the acceptor (i.e. the F8BT) will be preferentially orientated along a parallel direction to the donor dipoles (the InGaN/GaN MQWs) due to enhanced nano-confinement of the F8BT chains along the grating orientation. Of course, a reduction in nano-channel width further strengthens the enhancement of the nano-confinement, increasing the efficiency of NRET from the InGaN/GaN MQWs to the F8BT polymer.

The NRET efficiency can be calculated using Equation 7^14^,^24^,^25^


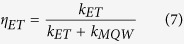


Figure 4d presents the NRET efficiency as a function of nano-channel width, demonstrating that the NRET efficiency increases with decreasing nano-channel width. The calculation of the NRET efficiency has been carried out based on an approach introduced in refs 11 and 23. It is well-known that not all the MQW region contributes to the NRET process and that the NRET process can take place effectively only in the region with a 10 nm separation between the donor (InGaN MQWs) and the acceptor (F8BT) *dipoles. Consequently, as studied previously, a correction factor needs to be included in order to calculate the NRET efficiency*^11^,^14^. In detail, the NRET efficiency increases from approximately 40% to 90%, giving an enhancement factor of more than 2 times. It is worthwhile noting that the NRET process can still occur for the randomly distributed F8BT dipoles, but in a very inefficient way as a result of a reduced dipole orientation factor, namely, the average dipole factor (i.e., *k*^2^ = 2/3).

In conclusion, highly polarized white light emission has been achieved from our hybrid organic/III-nitride devices. The hybrid devices have been fabricated by means of developing a novel nanofabrication technique, where a 1D grating structure has been fabricated into a blue InGaN/GaN MQW structure and yellow emitting F8BT as a down conversion material is filled into the nano-channels that form the 1D grating under an alignment enhancement process. The InGaN/GaN based 1D grating structure serves as both blue lighting source and polarizer. The yellow emission from the F8BT filled into the nano-channel exhibits polarised properties, strongly depending on the nano-channel width. Consequently, our hybrid device emits polarized white with an overall polarisation degree of up to 41.5%. Furthermore, as a result of the utilisation of the grating structure, the NRET efficiency between the InGaN/GaN MQWs and the yellow polymer has been significantly enhanced by a factor of up to almost 2. When the nano-channel width is below 100 nm, the highest NRET efficiency of up to 90% has been achieved, where the relative orientation between the dipoles in the donor and diploes in the acceptor leads to a maximised contribution to the NRET efficiency.

## Methods

### Fabrication of one-dimensional (1D) grating structures

A standard InGaN/GaN MQW epi-wafer was used to fabricate the grating structures. The epiwafer was grown by MOCVD on a double side polished sapphire substrate using our high temperature AlN buffer approach. After a high temperature annealing process on sapphire, an initial 200 nm AlN buffer layer was grown, followed by a 1.2 μm GaN buffer, then 5 pairs of In_0.20_Ga_0.80_N: 2,5 nm/GaN:10 nm MQWs, and finally a 10 nm GaN capping layer. A 250 nm thick silicon dioxide (SiO_2_) was first deposited on the top of the epi-wafer by plasma enhanced vapour deposition (PECVD). Afterwards, a thin photoresist film (650 nm) was then deposited by spin coating. The grating patterns were then written on the photoresist by a confocal microscope system, equipped with a 375 nm continuous wave (CW) diode laser, a 100× objective lens with an NA of 0.95 and a high resolution xyz piezo-scanning stage. The grating patterns were written in an area of 70 × 70 μm on the photoresist. A standard reactive ion etching (RIE) using *CHF*_*3*_*/Ar* gasses was then used to transfer the patterns into the *SiO*_*2*_ as a second mask for further etching through the InGaN MQWs by inductively coupled plasma etching (ICP) with *Cl*_*2*_*/Ar* gasses in order to form the final 1D grating structures.

### Fabrication of one-dimensional (1D) hybrid devices

For the fabrication of the hybrid devices, the poly (9, 9-dioctylfluorene-alt-benzothiadiazole), known as F8BT, organic polyfluorene co-polymer was dissolved in toluene in a 5 mg/mL concentration. The F8BT was then spin coated on the top of the grating structures to fill the periodic nano-channels. In order to align the F8BT chains, the samples were sandwiched between two aluminum plates which are bolted together to apply a high pressure on the samples and then placed on a hot plate at 160 °C for 1 hour in order to transition to a liquid crystal phase and effectively align the F8BT along the nano-channels. Finally, the samples were slowly cooled down to 70 °C, where the chains are “frozen” and then the pressure on the samples were then released. All processing of the F8BT was carried out in an anaerobic glovebox.

**Polarisation dependent PL measurements** were carried out in an in-house made micro-PL system. A 375 nm pulsed laser diode was used to selectively excite the InGaN/GaN MQWs, illuminating through the backside of the samples. An objective lens featured with 50x magnification and 0.43 NA is used, allowing us to focus the laser beam down to ~2 μm in diameter. The system is also equipped with a 1 mm fibre bundle to collect the emission. A 0.55 m Jobyn Yvon spectrometer (iHR550) was used to disperse the emission, equipped with an air-cooled charge coupled device (CCD). A polariser was placed in front of the collection fibre. The polarisation of the system has been removed from our measurements by normalising the hybrid emission spectra over the emission of a halogen lamp used as calibration light source. All of the measurements were performed at room temperature, where the samples were kept in an oxygen free cryostat under high vacuum (~10^−6^ Torr) to minimise the photo-oxidation induced degradation of the F8BT.

**For Time-resolved micro photoluminescence (μ-TRPL)** measurements, the above micro-PL system equipped with a time-correlated single photon counting (TCSPC) system was employed. This system is also equipped with a 375 nm pulsed diode laser with a 50 picoseconds (ps) pulse width as an excitation source. A standard monochromator and a Hamamatsu hybrid photon counting PMT are used to disperse and then detect luminescence, respectively. The overall system response-time is 120 ps, and the average excitation power used is 0.15 mW at a pulse repetition rate of 10 MHz. The identical objective lens described above was used to focus the laser beam.

## Additional Information

**How to cite this article**: Athanasiou, M. *et al*. Polarized white light from hybrid organic/III-nitrides grating structures. *Sci. Rep.* **7**, 39677; doi: 10.1038/srep39677 (2017).

**Publisher's note:** Springer Nature remains neutral with regard to jurisdictional claims in published maps and institutional affiliations.

## Supplementary Material

Supplementary Information

## Figures and Tables

**Figure 1:**
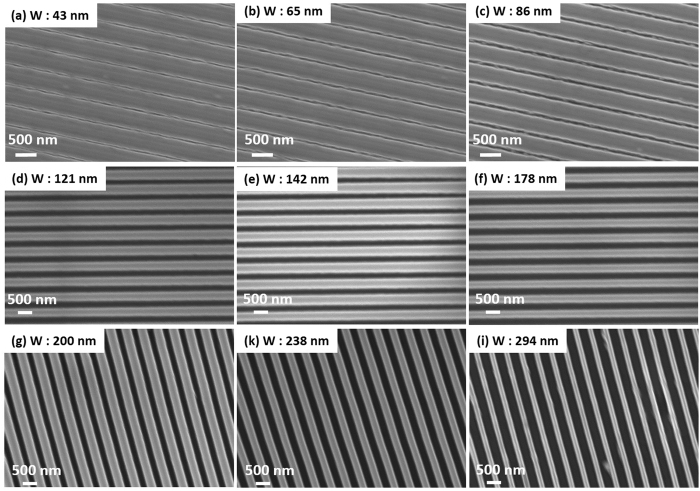
(**a–i**) Top-view scanning electron microscopy (SEM) images of the grating structures with a nano-channel width of 43, 65, 86, 121, 142, 178, 200, 238 and 294 nm, respectively. The period of the gratings (i.e., the centre to centre separation) was fixed at 510 nm.

**Figure 2:**
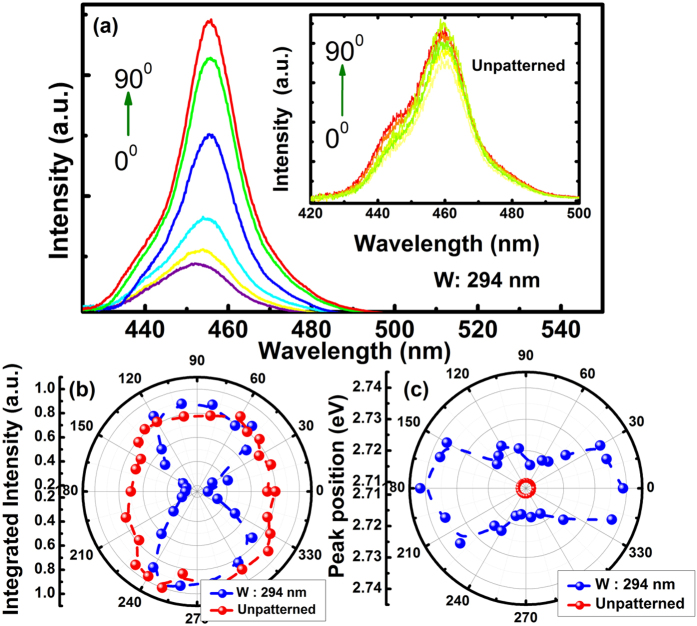
(**a**) Micro-PL measurements as a function of polarisation angle for a grating structure with a nano-channel width of 294 nm. Inset shows the polarisation dependent PL spectra of the unpatterned sample for a comparison; (**b**) Integrated PL intensities for the grating structure and the un-patterned sample plotted in the polar co-ordinate system showing an asymmetric behaviour, the finger print for a polarised emission from the grating structure, while the un-patterned sample shows a symmetric behaviour indicating an un-polarised emission; and (**c**) Emission wavelength plotted in the polar coordinate system for both the un-patterned sample and the grating structure.

**Figure 3:**
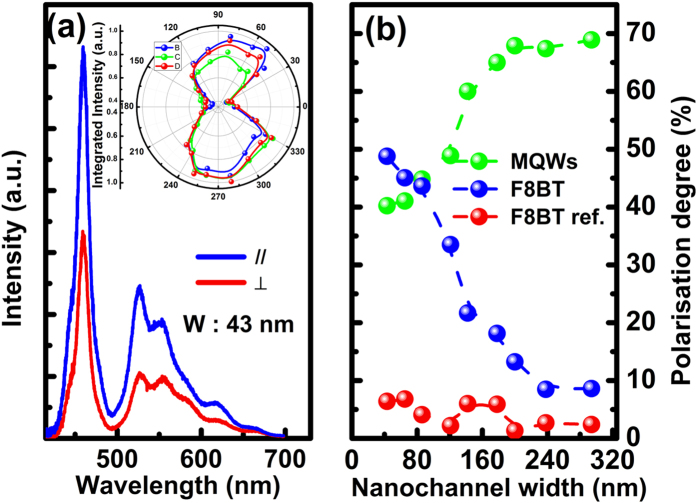
(**a**) Typical hybrid spectra of the grating structure measured at two extreme polarisation angles, namely, the polariser is set to be parallel and perpendicular to the grating orientation. Inset shows integrated PL intensity as a function of polarisation angles plotted in the polar co-ordinate system for the InGaN MQWs emission, F8BT emission and the overall emission; and (**b**) Polarisation degree of the InGaN MQWs, F8BT polymer and the reference sample plotted as a function of nano-channel width.

**Figure 4:**
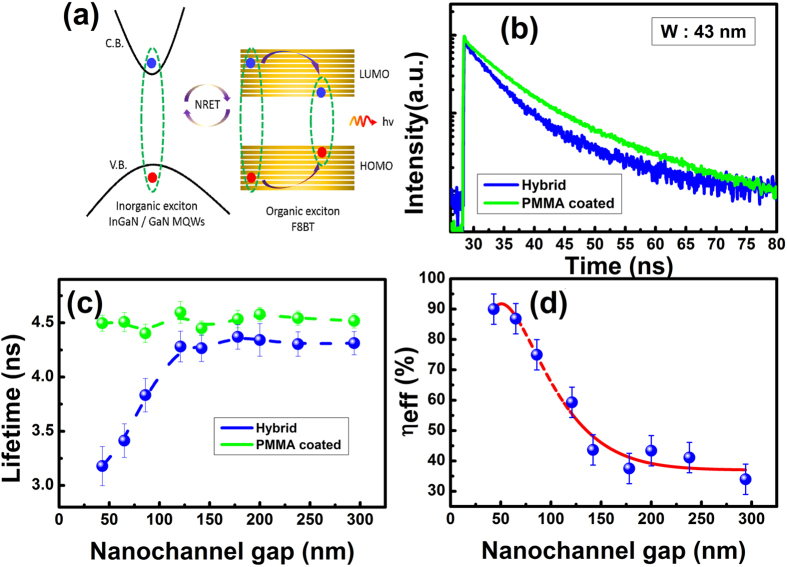
(**a**) Schematic illustration of the NRET process in a hybrid organic/inorganic device; (**b**) Time-decay PL traces for the grating structures with the F8BT and PMMA filled into the nano-channel, respectively, measured at 460 nm (i.e., the emission wavelength from the InGaN/GaN MQWs at RT; (**c**) Decay lifetime of the InGaN MQWs in the F8BT filled structure and the PMMA filled structure as a function of nano-channel width, where a dramatic reduction in PL decay lifetime has been observed with reducing nano-channel width when the nano-channel width is below 100 nm; and (**d**) NRET efficiency of our hybrid devices as a function of nano-channel width.
