# Supplementary material for: Polarized white light from hybrid organic/III-nitrides grating structures
Source: Sci Rep. 2017 Jan 3;7:39677. doi: 10.1038/srep39677 (PMC5206735; doi:10.1038/srep39677)
Supplement: Supplementary Information [file srep39677-s1.pdf]

Polarized white light from hybrid organic/III-nitrides grating structures

M. Athanasiou, R. M. Smith, S. Ghataora and T. Wang*

Department of Electronic and Electrical Engineering, University of Sheffield, United Kingdom

* Corresponding author: t.wang@sheffield.ac.uk

Supplementary Information

Our InGaN/GaN multiple quantum wells (MQWs) based one-dimensional (1D) grating structures were fabricated with a WiTEC confocal microscope using the direct laser writing technique. The procedure is schematically depicted in **Fig. S1:** (a) InGaN / GaN MQWs grown on a double side polished sapphire substrate; (b) A 250 nm thick SiO_2 layer deposited directly on top of the sample by standard plasma enhanced chemical vapour deposition (PECVD); (c) Photoresist was deposited by spin coating at 6000 rpm for 30 seconds, giving a layer thickness of approximately 600 nm, followed by a UV light exposure and development; (d) Grating patterns were written by confocal microscope laser exposure using a 375 nm laser diode followed by an oxygen plasma cleaning process to remove the residue from the trenches; (e) Reactive ion etching (RIE) was then employed to etch the grating pattern into the secondary SiO_2 mask, which acts as hard mask for further etching; (f) Inductively coupled plasma (ICP) etching was then used to further etch the gratings into the InGaN / GaN MQWs. The depth of the gratings is approximately 400 nm into the GaN through the InGaN MQWs.

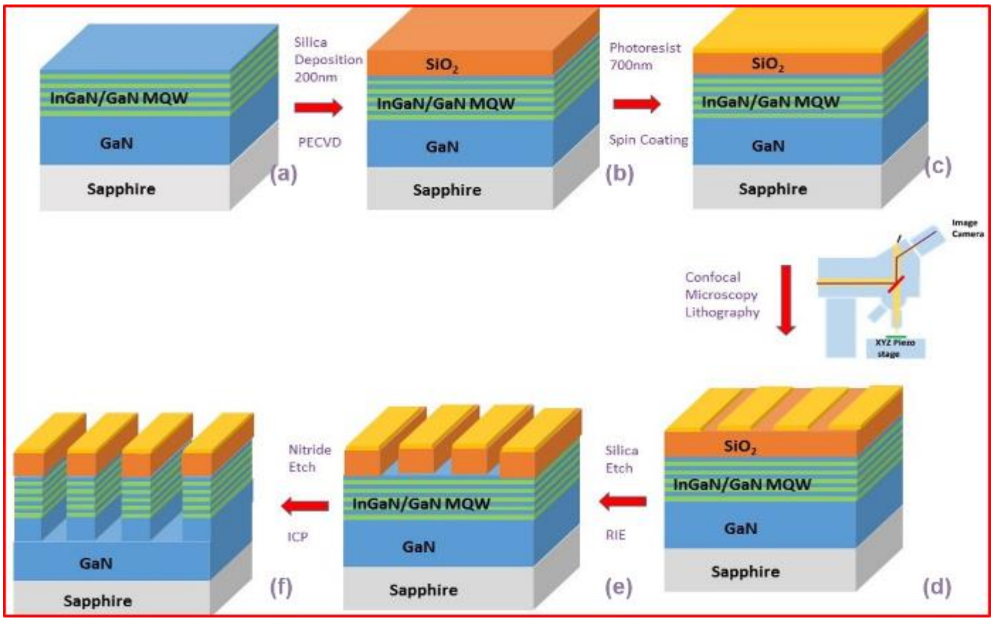

Figure S1 *Schematic illustration of the fabrication procedure for the 1D – grating structures in InGaN / GaN MQWs using confocal laser lithography technique.*

Each step of fabrication has been monitored in order to ensure the high quality of fabrication as shown in **Fig. S2:** (a) Optical microscope image of the gratings written directly on photoresist; (b) *SEM* image of the *SiO2 h*ard mask gratings after the *RIE* etching; (c) High magnification SEM image of the gratings with 510 nm centre to centre separation revealing the straight sidewall edges; (d) Low magnification SEM image showing the a 70 x 70 µm square grating patterns.

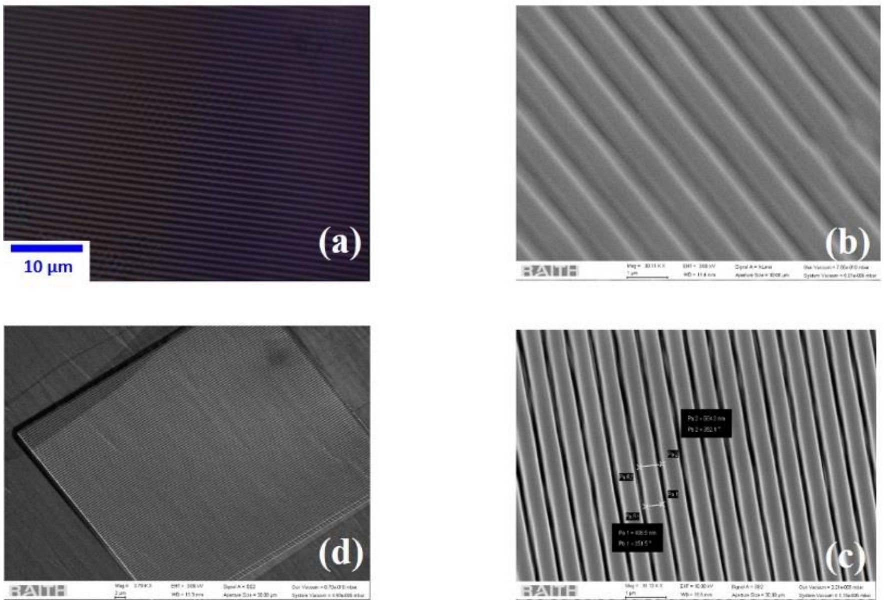

Figure S2 *Optical and SEM images of the grating structures taken at each step of the fabrication procedure to ensure a high quality of fabrication.*

For the fabrication of the hybrid grating structures a schematic illustration of each step is shown in **Fig. S3:** (a) F8BT was first dissolved in toluene at a concentration of *5 mg / mL* and spin coated on top of the gratings at a speed of 4000 rpm for 30 seconds to fill in the gaps. The whole process, including the solution preparation and deposition of the F8BT has been carried out in a glovebox, in an oxygen free environment to protect the polymer from oxidation; (b) The sample was then sandwiched between two metal plates which were bolted together in order to apply a high pressure. At the same time, the sample was heated up to $160\ ℃$ for 1 hour, which is above the glass transition temperature ($T_g \sim 90\ ℃$), in order to allow the F8BT polymer to transition to its liquid crystal phase and force it to align with the underlying gratings; (c) The sample was then slowly cooled down to $70\ ℃$, thus allowing the F8BT chains to freeze, forcing them to remain aligned with the grating structures. The pressure was then released and the samples were transferred to a vacuum chamber for optical characterisation under vacuum to avoid photo-oxidation of the polymer.

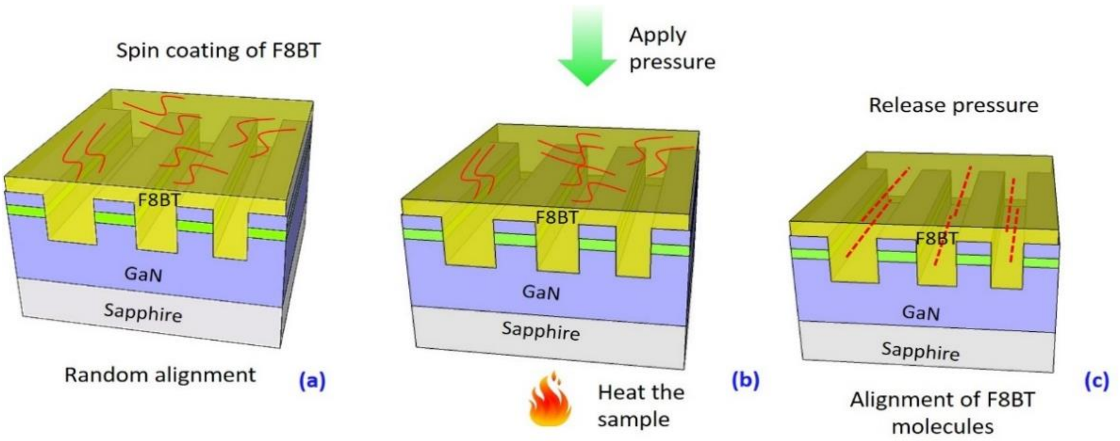

***Figure S3** Schematic illustration of the F8BT chain alignment process for the polarised hybrid grating structures.*

Absorption and PL emission spectra of F8BT along with the InGaN MQW PL spectrum are plotted in **Fig. S4.** A great overlap can be seen between the absorption spectrum of the acceptor dipole (F8BT) and the emission spectrum of the donor dipole (InGaN MQWs), thus helping to maximise the NRET rate between the two dipoles.

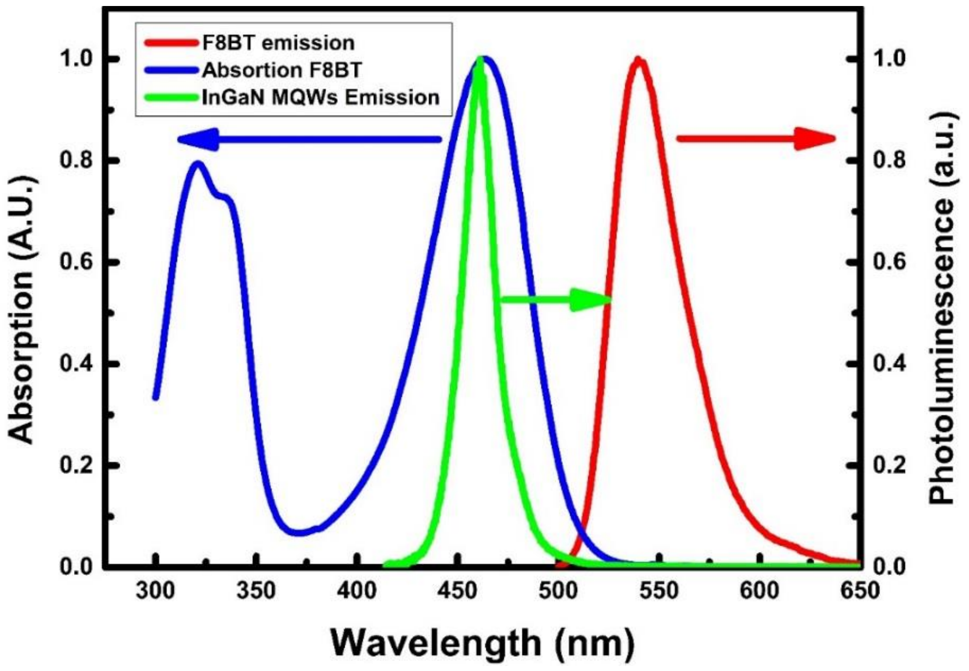

***Figure S4** Absorption and emission spectra of the F8BT and InGaN MQWs.*

Fig. S5 Shows the µ-PL spectra of the unpatterned sample and all of the grating samples measured at both orthogonal polarisations when the polariser is set to parallel and perpendicular with the grating structures.

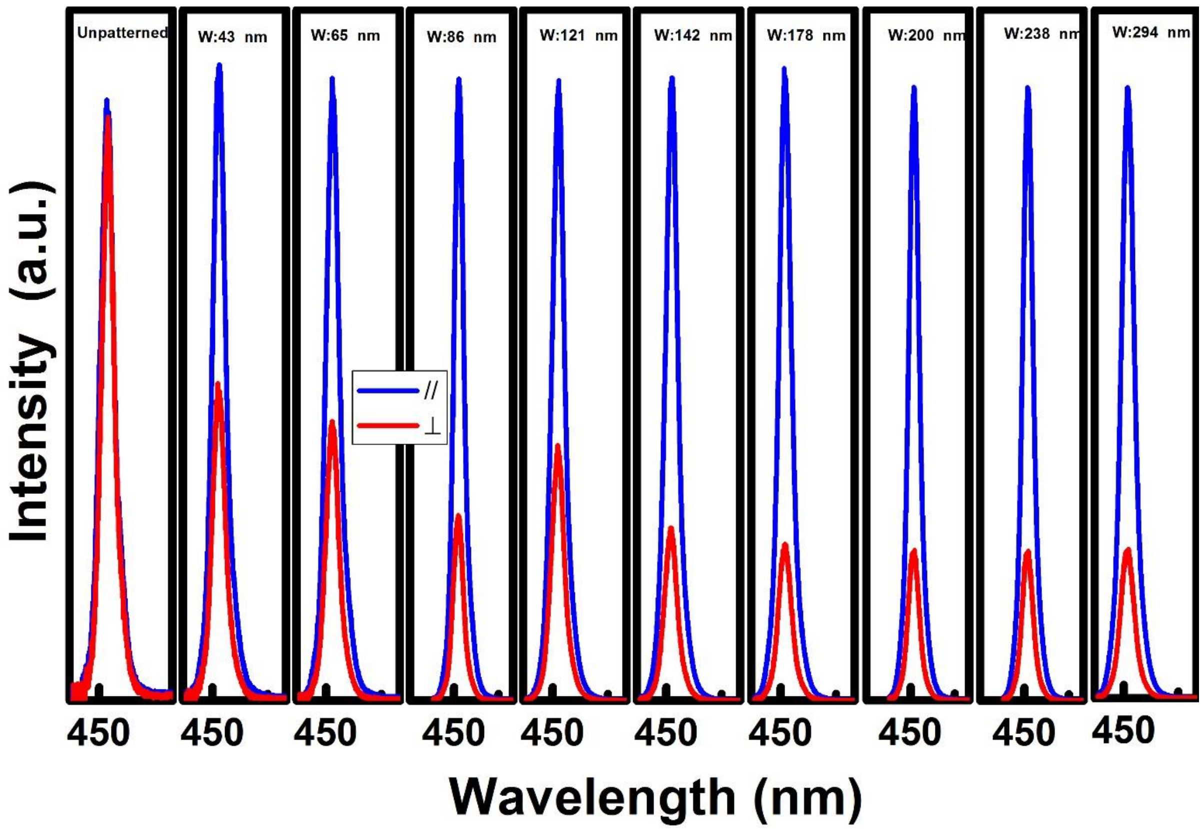

Figure S5 *µ-PL spectra of InGaN MQWs for the unpatterned and grating structures taken with the polariser set at the two orthogonal positions; parallel and perpendicular to the gratings.*

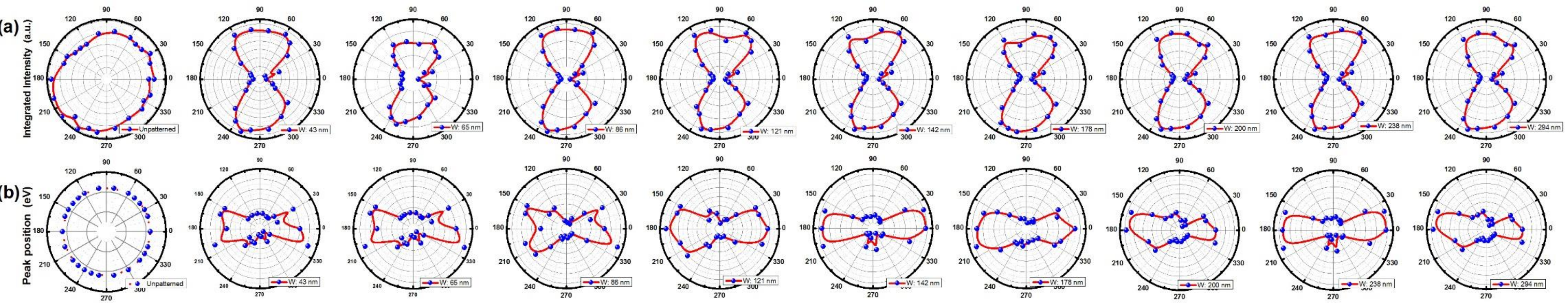

Figure S6 *Polar plots of the integrated PL intensity and the peak position of the InGaN MQWs for the unpatterned and the grating structures.*

Fig. S6 Polar plots of the integrated PL intensities of the InGaN / GaN MQWs plotted as a function of the polariser angle for unpatterned and grating samples; (b) Peak positions of the unpatterned and grating samples plotted in polar plots against the polariser angle.

Fig. S7 Shows the µ-PL spectra of the unpatterned and all of the hybrid grating samples measured at both orthogonal polarisations when the polariser is set to parallel and perpendicular with the gratings structures

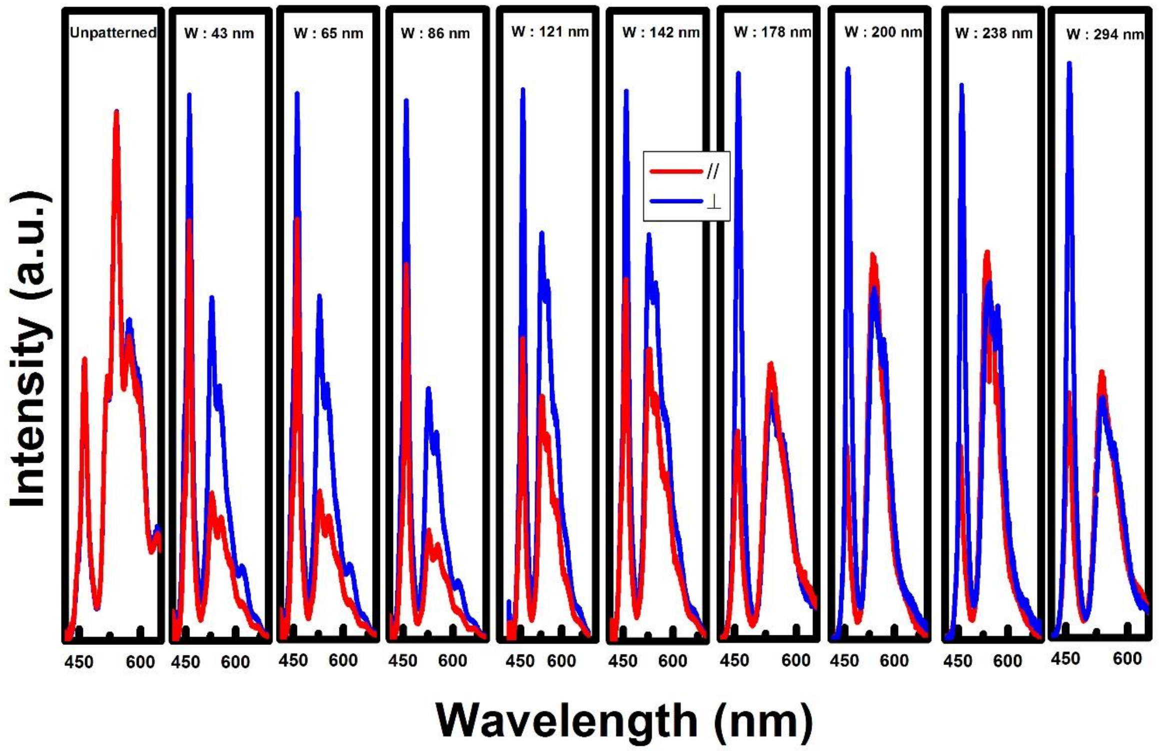

Figure S7 *µ-PL spectra of the hybrid InGaN MQWs / F8BT samples subjected to the F8BT alignment process described earlier in* ***Fig. S3.*** *The spectra of the unpatterned and grating structures were taken with the polariser set at the two orthogonal positions; parallel and perpendicular to the gratings structures.*

Fig. S8 Shows the µ-PL spectra of the unpatterned and all of the reference hybrid grating samples without pressure and heat applied measured at the both orthogonal polarisations when the polariser is set to parallel and perpendicular with the grating structures. No polarisation of the F8BT can be detected due to the random alignment of the F8BT chains in macroscopic domains.

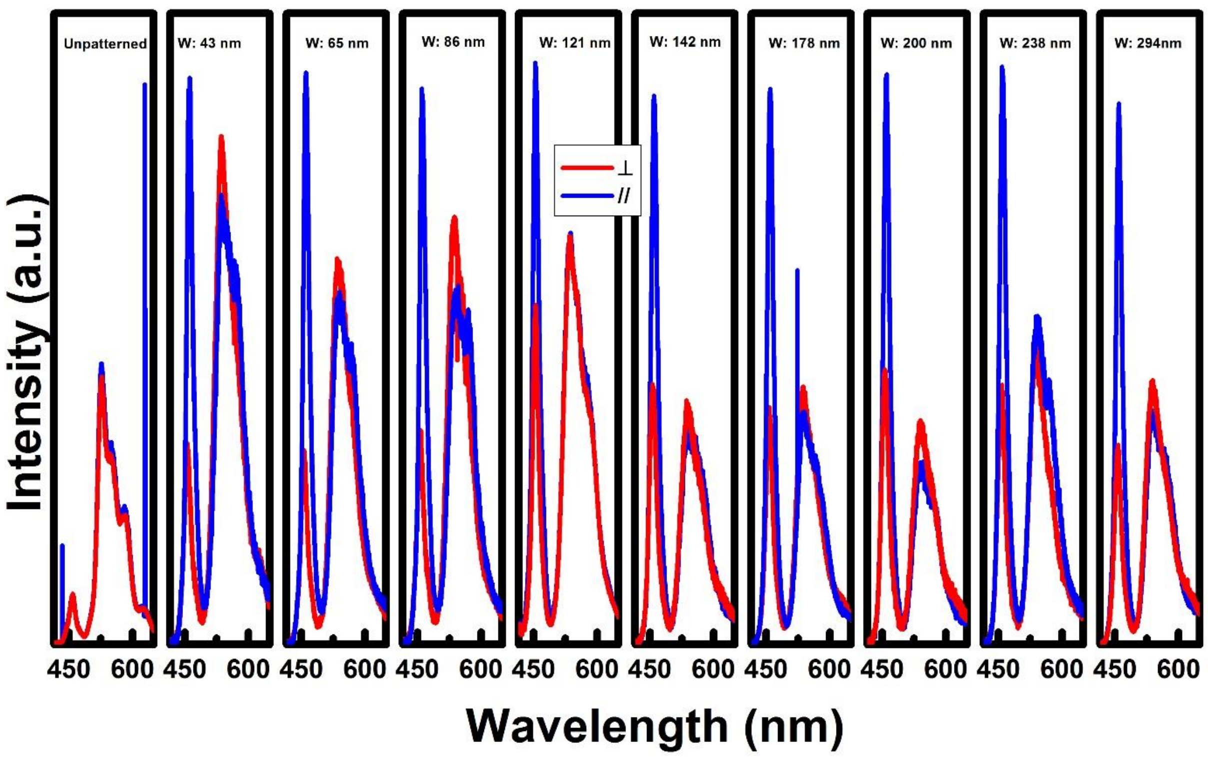

Figure S8 show *μ-PL spectra of the hybrid InGaN MQWs / F8BT samples without being subjected to the F8BT to the alignment process. The spectra of the unpatterned and grating structures were taken with the polariser set at the two orthogonal positions; parallel and perpendicular to the grating structures.*
